# Kagami–Ogata syndrome: a case report

**DOI:** 10.1186/s13256-022-03512-6

**Published:** 2022-07-22

**Authors:** Tharindi Suriapperuma, Shobhavi Randeny, Sachith Mettananda

**Affiliations:** 1 Department of Paediatrics, Faculty of Medicine, University of Kelaniya, Colombo, Sri Lanka; 2 Colombo North Teaching Hospital, Ragama, Sri Lanka

**Keywords:** Kagami–Ogata syndrome, Genetic imprinting disorder, Coat-hanger angle, Mid-to-widest thoracic diameter

## Abstract

**Background:**

Kagami–Ogata syndrome is a rare genetic imprinting disorder involving the 14q32.2 genomic location of chromosome 14. The estimated incidence is less than 1 per 1 million. Here we report a male neonate with Kagami–Ogata syndrome presenting with severe respiratory distress requiring mechanical ventilation since birth.

**Case presentation:**

A Sri Lankan male neonate born at term via caesarean section to a mother with type 1 diabetes mellitus and hypothyroidism developed respiratory distress immediately after birth. On examination, the baby had facial dysmorphism with a hirsute forehead, full cheeks, flat nasal bridge, elongated protruding philtrum, and micrognathia. His chest was small and bell shaped, and he had severe intercostal and subcostal recessions. His abdominal wall was lax and thin, with evidence of divarication of the recti. Bowel peristalsis was easily visible through the abdominal wall. The chest x-ray showed narrowing of the rib cage with crowding of the ribs in a “coat-hanger” appearance. The coat-hanger angle was 32°, and the mid-to-widest thoracic diameter was 68%. On the basis of facial dysmorphism, chest and anterior abdominal wall abnormalities, coat-hanger appearance of the rib cage, increased coat-hanger angle, and reduced mid-to-widest thoracic diameter, a clinical diagnosis of Kagami–Ogata syndrome was made. Owing to severe respiratory distress, the baby required intubation and ventilation immediately after birth. He was ventilator-dependent for 3 weeks; however, he was successfully weaned off the ventilator on day 22 after several failed extubation attempts. At 3-month follow-up, he had generalized hypotonia and mild global developmental delay. His developmental age corresponded to 2 months.

**Conclusions:**

We report a patient with Kagami–Ogata syndrome presenting with respiratory distress immediately after birth. This case report highlights the importance of being aware of this rare condition, which could present as severe respiratory distress in term and preterm newborns. A positive diagnosis could avoid unnecessary treatment and aid in accurate prognostication.

## Background

Kagami–Ogata syndrome is a rare genetic imprinting disorder involving genes of the 14q32.2 region of chromosome 14. The majority of cases, approximately two-thirds, are due to paternal uniparental disomy 14 [upd(14)pat]. The estimated incidence is less than 1 in 1 million, and only about 80 patients have been reported so far. The characteristic features include a small bell-shaped thorax with a coat-hanger appearance of the ribs, abdominal wall defects, placentomegaly, and polyhydramnios [1]. Here we report a male neonate with Kagami–Ogata syndrome presenting with severe respiratory distress requiring mechanical ventilation since birth.

## Case presentation

A male newborn was admitted to the neonatal intensive care unit owing to respiratory distress that appeared immediately after birth. He was born at term via an elective caesarean section to a mother with type 1 diabetes mellitus and hypothyroidism. Apgar scores were 9, 9, and 10 at 1, 5, and 10 minutes, respectively. The anthropometric parameters at birth were: weight, 2.55 kg; length, 48 cm; and occipitofrontal circumference, 33 cm.

**Fig. 1:**
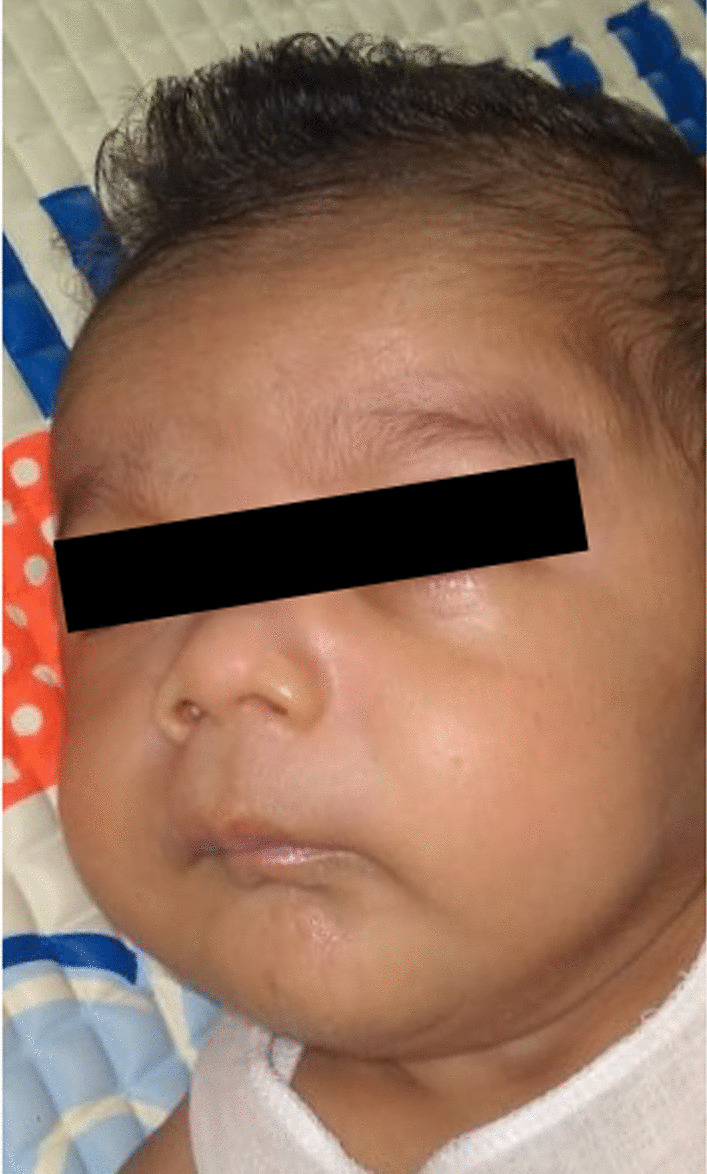
Photograph showing subtle facial dysmorphism with a hirsute forehead, full cheeks, flat nasal bridge, elongated protruding philtrum, and micrognathia

On examination, the baby had subtle facial dysmorphism with a hirsute forehead, full cheeks, flat nasal bridge, elongated protruding philtrum, and micrognathia (Fig. 1). His chest was small and bell shaped, and he had severe respiratory distress with intercostal and subcostal recessions. His abdominal wall was lax and thin, with evidence of divarication of the recti. Bowel peristalsis was easily visible through the abdominal wall; however, there was no umbilical or inguinal hernia. The cardiovascular system was clinically normal.

**Fig. 2:**
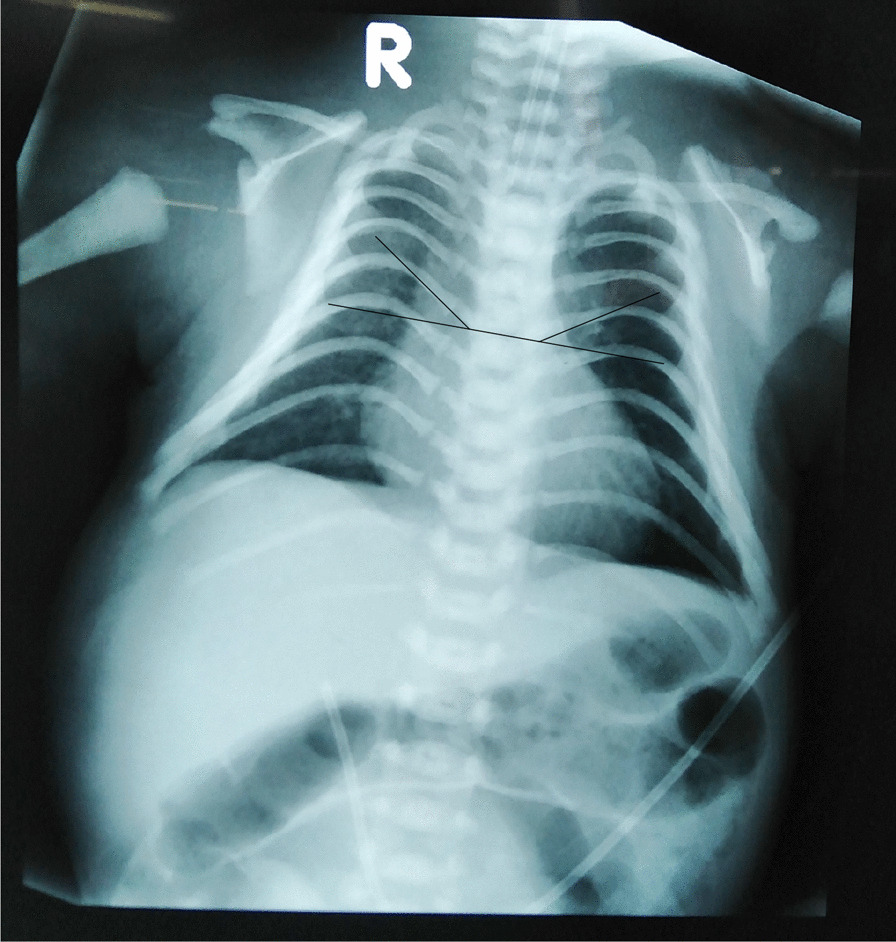
Chest x-ray showing coat-hanger appearance of the rib cage with increased coat-hanger angle (33° on the right side and 31° on the left side). In addition, the ratio between mid-to-widest thoracic diameter is significantly decreased to 68%

The chest x-ray showed narrowing of the rib cage with crowding of the ribs in a “coat-hanger” appearance (Fig. 2). The coat-hanger angle (CHA) was 32°, and the mid-to-widest thoracic diameter was 68%. There was no radiological evidence of surfactant deficiency, pneumonia, or congenital malformations of the lungs. Basic biochemical, microbiological, and hematological investigations were normal. The echocardiogram revealed a small ostium secundum atrial septal defect. The skeletal survey did not reveal abnormalities in long bones.

On the basis of facial dysmorphism, chest and anterior abdominal wall abnormalities, coat-hanger appearance of the rib cage, increased CHA, and reduced mid-to-widest thoracic diameter, the clinical diagnosis of Kagami–Ogata syndrome was made. His karyotype was normal, and further genetic testing was not done owing to unavailability.

**Fig. 3:**
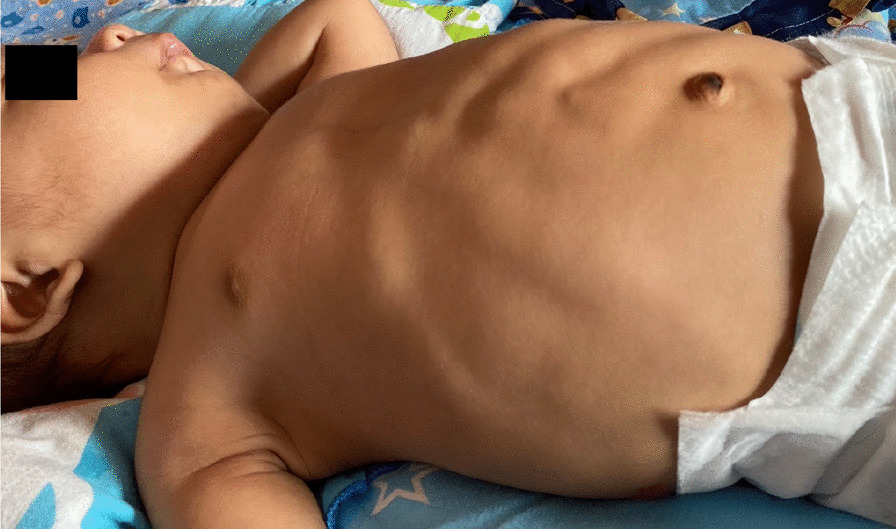
Photograph taken at 3 months showing persistently lax abdominal muscles with prominent peristalsis

Owing to severe respiratory distress, the baby required intubation and mechanical ventilation immediately after birth. He remained ventilator-dependent for 3 weeks; however, he was successfully weaned off the ventilator on day 22 after several failed extubation attempts. Feeding was initiated with expressed breast milk owing to poor sucking, and the baby was commenced on early intervention to improve neurological outcome. At 3-month follow up, he had generalized hypotonia, lax abdominal muscles with prominent peristalsis, and mild global developmental delay with the developmental age corresponding to 2 months (Fig. 3).

## Discussion and conclusion

Kagami–Ogata syndrome is a rare genetic disorder that was first described by Wang and others in 1991 [2]. It is a genomic imprinting disorder involving the 14q32.2 genomic location on chromosome 14 [1]. The majority of cases, approximately two-thirds, are due to paternal uniparental disomy 14 [upd(14)pat], which could occur as *de novo* mutations, paternally inherited Robertsonian translocations, or a monosomy rescue leading to paternal isodisomy. The other genetic mechanism causing Kagami–Ogata syndrome is microdeletion of the differentially methylated region at 14q32.2 of the maternal chromosome [1, 3].

Patients with Kagami–Ogata syndrome present with respiratory distress at birth due to the small narrow bell-shaped thorax and the coat-hanger appearance of the rib cage [1, 2]. The measurement of CHA as a radiological feature of Kagami–Ogata syndrome was introduced by Miyazaki and others in 2011. The CHA is the average value of the angles between the peak point (or the center in the absence of a peak point) of the sixth posterior rib and the horizontal axis on both sides [4]. If CHA is more than 25°, Kagami–Ogata syndrome should be strongly suspected [1]. In our patient, the CHA was 32°. Another radiological feature of Kagami–Ogata syndrome is the decreased ratio of mid-to-widest thoracic diameter [2]. In our patient, the mid-to-widest thoracic diameter ratio was significantly decreased and was 68%.

Abdominal wall defects such as omphalocele, hernia, and divarication of recti are reported in patients with Kagami–Ogata syndrome [1, 5]. Our patient had lax and thin abdominal wall with visible peristalsis and rectus divarication. Facial dysmorphism such as frontal bossing, hirsute forehead, depressed nasal bridge, micrognathia, retrognathia, full cheeks, webbed neck, short palpebral fissures, protruding philtrum, small ears, and anteverted nares are found to be characteristic of Kagami–Ogata syndrome [1, 2].

The confirmation of Kagami–Ogata syndrome is based on molecular genetic testing. However, these tests are not widely available. The specific genetic tests include methylation studies and microsatellite analysis for uniparental disomy 14 [1]. Owing to the unavailability and limitation of facilities, molecular genetic tests were not done on our patient.

The most important take-home message of this case report is that pediatricians and neonatologists should be aware of this rare condition that presents with respiratory distress in term and preterm neonates. The lack of awareness could lead to diagnostic uncertainties. In the index patient, the diagnosis was suspected by the characteristic chest x-ray appearance on the first day of life. It avoided unnecessary treatment such as surfactant and second-line antibiotics, despite having multiple failed extubation attempts, which could be attributed to the condition.

In conclusion, we report a patient with Kagami–Ogata syndrome presenting with respiratory distress immediately after birth. This case report highlights the importance of being aware of this rare condition, which could present as severe respiratory distress in term and preterm newborns. A positive diagnosis could avoid unnecessary treatment and aid in accurate prognostication.

## Data Availability

Not applicable.

## Abbreviation

CHA: Coat-hanger angle

## References

[1] Sakaria RP, Mostafavi R, Miller S, Ward JC, Pivnick EK, Talati AJ. Kagami–Ogata syndrome: case series and review of literature. AJP Rep. 2021;11(2):e65–75. 10.1055/s-0041-1727287 PMC8159623 34055463

[2] Ogata T, Kagami M. Kagami–Ogata syndrome: a clinically recognizable upd(14)pat and related disorder affecting the chromosome 14q32.2 imprinted region. J Hum Genet. 2016;61(2):87–94. 10.1038/jhg.2015.113 PMC4771937 26377239

[3] Wang X, Pang H, Shah BA, Gu H, Zhang L, Wang H. A male case of Kagami–Ogata syndrome caused by paternal unipaternal disomy 14 as a result of a Robertsonian translocation. Front Pediatr. 2020;8:88. 10.3389/fped.2020.00088 PMC7076151 32211354

[4] Miyazaki O, Nishimura G, Kagami M, Ogata T. Radiological evaluation of dysmorphic thorax of paternal uniparental disomy 14. Pediatr Radiol. 2011;41(8):1013–9. 10.1007/s00247-011-2132-1 21607596

[5] Huang H, Mikami Y, Shigematsu K, Uemura N, Shinsaka M, Iwatani A, Miyake F, Kabe K, Takai Y, Saitoh M, *et al*. Kagami–Ogata syndrome in a fetus presenting with polyhydramnios, malformations, and preterm delivery: a case report. J Med Case Rep. 2019;13(1):340. 10.1186/s13256-019-2298-y PMC6873543 31753000

